# Prolonged outbreak of clonal MDR *Pseudomonas aeruginosa* on an intensive care unit: contaminated sinks and contamination of ultra-filtrate bags as possible route of transmission?

**DOI:** 10.1186/s13756-016-0157-9

**Published:** 2016-12-06

**Authors:** Florian Salm, Maria Deja, Petra Gastmeier, Axel Kola, Sonja Hansen, Michael Behnke, Désirée Gruhl, Rasmus Leistner

**Affiliations:** 1 Institute of Hygiene and Environmental Medicine, National Reference Centre for the Surveillance of nosocomial Infections, Charité Universitaetsmedizin Berlin, Hindenburgdamm 27, D-12203 Berlin, Germany; 2 Department of Anesthesiology and Intensive Care, Charité Universitaetsmedizin Berlin, Campus Benjamin Franklin, Hindenburgdamm 30, 12203 Berlin, Germany

**Keywords:** Outbreak, Intensive care unit, Pseudomonas aeruginosa, Health care-associated infection

## Abstract

**Background:**

We report on an outbreak in a surgical, interdisciplinary intensive care unit (ICU) of a tertiary care hospital. We detected a cluster of ICU patients colonized or infected with multidrug-resistant *Pseudomonas aeruginosa*. We established an outbreak investigation team, performed an exploratory epidemiological analysis and initiated an epidemiology-based intervention.

**Methods:**

As part of the outbreak investigation, we performed microbiological examinations of the sinks in the patient rooms and a retrospective case-control study. All patients admitted to the outbreak ICU between January 2012 and February 2014 were included. Cases were patients colonized with the outbreak strain. Controls were patients with a different *Pseudomonas aeruginosa* strain. Risk factors were evaluated using multivariable conditional logistic regression analysis. Strain typing was performed using the repetitive element-based polymerase chain reaction (rep-PCR) DiversiLab system.

**Results:**

The outbreak strain was found in the sinks of five (of 16) patient rooms. Altogether 21 cases and 21 (randomly selected) controls were included. In the univariate analysis, there was no significant difference in baseline data of the patients. In the multivariate analysis, stay in a room with a colonized sink (Odds Ratio[OR] 11.2, *p* = 0.007) and hemofiltration (OR 21.9, *p* = 0.020) were independently associated with an elevated risk for colonization or infection by the outbreak strain. In a subsequent evaluation of the work procedures associated with hemofiltration, we found that the ultra-filtrate bags had been on average five times per day emptied in the sinks of the patient rooms and were used multiple for the same patient. We exchanged the traps of the contaminated sinks and eliminated work procedures involving sinks in patient rooms by implementation of single use bags, which are emptied outside patient rooms to reduce splash water at the sinks. In the 20 month follow-up period, the outbreak strain was detected only once, which indicated that the outbreak had been ceased (incidence 0.75% vs. 0.04%, *p* < 0.001) Furthermore, the incidence of *Pseudonomas aeruginosa* overall was significantly decreased (2.5% vs. 1.5%, *p* < 0.001).

**Conclusion:**

In ICUs, limiting work processes involving sinks results in reduced multidrug-resistant *Pseudomonas aeruginosa* rates. ICUs with high rates of *Pseudomonas aeruginosa* should consider eliminating work processes that involve sinks and potentially splash water in close proximity to patients.

**Trial registration:**

All data were surveillance based data which were obtained within the German Law on Protection against Infection (“Infektionsschutzgesetz”). Therefore a trial registration was not required.

## Background

Multidrug-resistant (MDR) *P. aeruginosa* are among the most commonly-found organisms which cause nosocomial infections in intensive care units [12, 13, 18, 21]. They are associated with increased mortality as well as increased hospital costs [14]. *P. aeruginosa* is a hydrophilic Gram-negative rod often found in water drainage systems in hospitals [17]. The organism has further been described as extremely adaptable to selective pressure caused by antimicrobial agents [13]. Due to the substantial necessity of antimicrobial therapy in intensive care units (ICU), the selective pressure in this setting is considerably high. This is supported by the finding that antimicrobial therapy prior to an ICU stay increases the risk for colonization with MDR *P. aeruginosa* [8, 15]*.* Once the organisms are introduced into an ICU, they can cause outbreaks that are often associated with sinks or faucets as a continuous source of further spread [1, 2, 11, 19, 20]. However, the most likely transmission route is direct person-to-person contact [10].

We report on an outbreak of MDR *P. aeruginosa* in an interdisciplinary ICU of a tertiary care, university hospital. The unit was comprised of 16 rooms with 30 intensive care beds. Within the scope of our infection control assignment, we collected all multidrug resistant organisms (MDRO) isolated from clinical or screening cultures and stored them in our MDRO bank. Whenever an epidemiological link between these MDRO isolates is suspected, molecular strain typing is performed. By this means, a cluster of MDR *P. aeruginosa* strains was observed in December 2013. Because the strain typing results suggested a clonal relationship, we subsequently re-cultured earlier MDR *P. aeruginosa* isolates from the frozen MDRO bank of our institute since January 2012. Furthermore, we decided to initiate an investigation to control the outbreak, find the transmission route, eliminate the potential source, and avoid future clusters.

## Methods

**Fig. 1:**
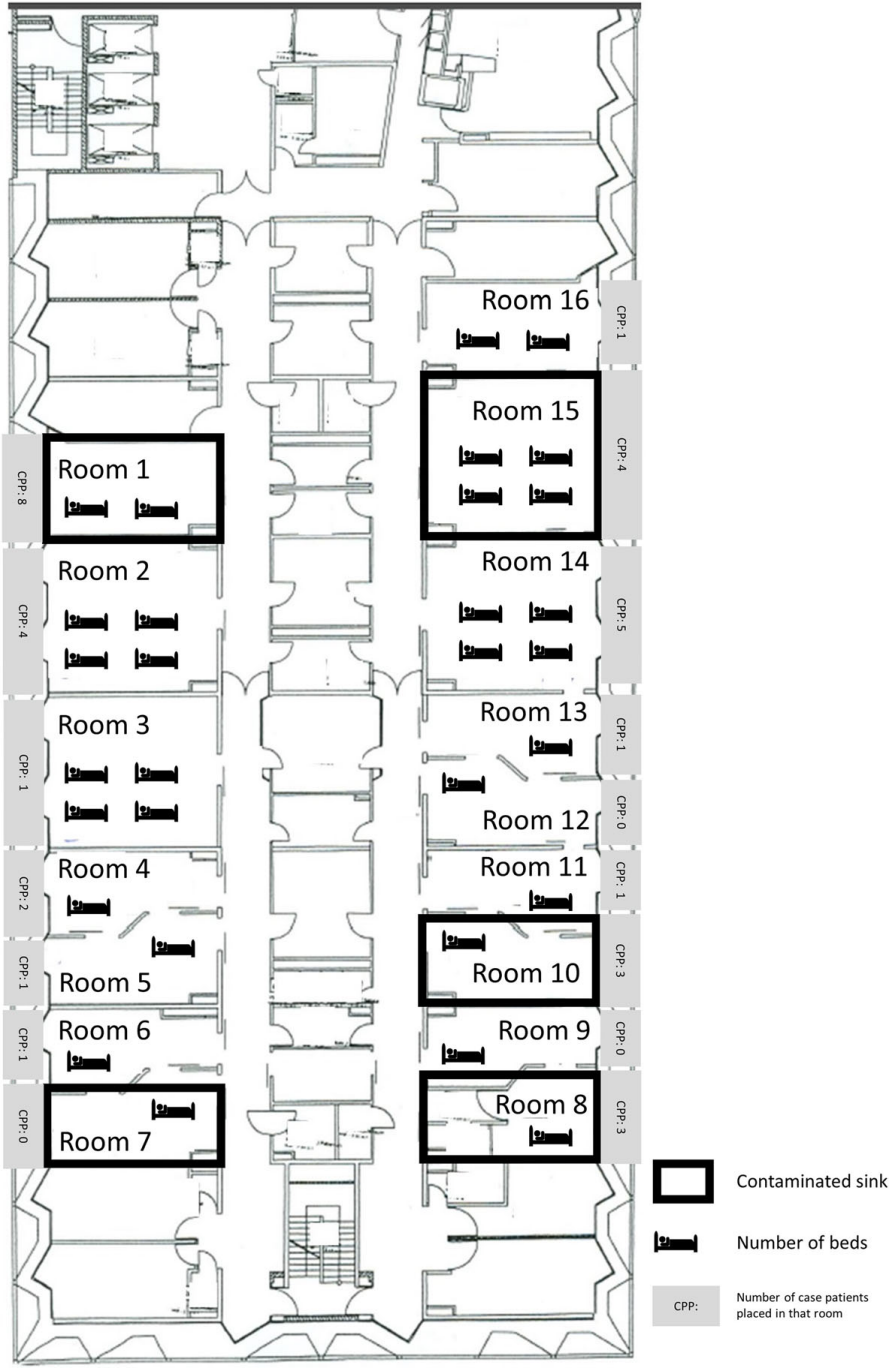
Overview of the outbreak ward including rooms with colonized sinks

The outbreak and the subsequent analysis was performed in a tertiary care university hospital with over 3000 beds. The outbreak ward was a surgical intensive care unit with 30 beds. All beds were equipped for intubation and ventilator support. The patient beds were distributed over 16 rooms. Each room was equipped with one clinical hand hygiene sink (Fig. 1). The sinks consisted of ceramic washbasin with chrome-plated tap made of metal and an attached downstream bacterial filter. Together with the rest of the room, the surfaces of the sinks were cleaned at least twice daily. The Sinks were also used for bathing and grooming the patients.

For the suspected outbreak, an outbreak investigation team was established comprised of two infection control physicians, one infection control nurse, the attending intensive care physician of the ward, and a microbiologist. Because all MDRO isolates were routinely collected and frozen, we were able to examine retrospectively all MDR *P. aeruginosa* isolates from the affected ward for the 2 years prior to the start of the outbreak investigation. *P. aeruginosa* isolates detected at least 72 h after admission to the ward were classified as ward-associated.

### Microbiological methods and clinical and environmental sampling

Clinical and environmental samples including identification on species level and antimicrobial susceptibility testing were performed by the VITEK 2 system (bioMérieux) and were interpreted according to European Committee on Antimicrobial Susceptibility Testing definitions (EUCAST, http://www.eucast.org). Phenotypical categorization of the outbreak strain as MDR (multidrug-resistant) was performed using standard definitions [13]. Rep-PCR was performed on all available clinical and environmental MDR *P. aeruginosa* isolates derived from the ICU between 1^st^ January 2012 and December 31, 2015. Isolates with rep-PCR profiles yielding a similarity of >95% were considered clonally related [6]. In order to detect outbreak clusters, we performed rep-PCR of all MDR *P. aeruginosa* isolates from clinical specimens of the respective ward between 1^st^ January 2012 and the intervention on April 30, 2014. As a follow up, the analysis was repeated until December 31, 2015 comparing new MDR *P. aeruginosa* strains to a reference outbreak strain from the initially discovered cluster. In order to assess the likelihood of environmental contamination and subsequent spread, the sinks of all patient rooms in the respective ward were probed using sterile cotton swabs. The MDR *P. aeruginosa* isolates collected environmentally were also compared to the reference outbreak rep-PCR profile. Rep-PCR was performed with the DiversiLab-System (BioMerieux) [5], using Pearson correlation coefficient pairwise pattern matching and the unweighted pair group method with arithmetic mean (UPGMA) clustering algorithm.

### Exploratory case-control-study

We conducted a retrospective case-control-study. The initial base cohort was comprised of all patients that were found (for the first time during their hospital stay) to be colonized or infected with *P. aeruginosa* 48 h after admission to the ICU. As a result these strains were considered ward-associated. All patients admitted to the outbreak ICU during the ongoing outbreak between 1^st^ January 2012 and 30^th^ April 2014 were included. Cases were defined as patients with either a colonization or infection by the MDR *P. aeruginosa* outbreak strain. Controls were patients colonized or infected with any *P. aeruginosa* strain other than the outbreak strain. The controls were randomly selected from the respective sub-cohort of all potential controls (*n* = 78). Risk factors for colonization were collected by means of a retrospective analysis of the patients’ records. Risk factors were extracted for the period between admission to the ward and *P. aeruginosa* detection. The following basic epidemiological parameters were collected: age, gender, overall length of stay on the ward, stay in a single or shared room as well as following risk factors for acquisition of the outbreak strain: surgical drainage from any body site, dialysis or hemofiltration, invasive intubation, central venous catheter (CVC), time (days) before *P. aeruginosa* detection, severity of diseases (SAPS II), stay in a room with a colonized sink, clinically diagnosed *P. aeruginosa* infection, tracheal cannula, leukopenia, administration of an immunosuppressive drug, shock as defined by intensive care medicine definitions [3], antimicrobial therapy, and contact with a patient colonized with the outbreak strain.

### Statistical methods

Differences were tested by Chi-square or Wilcoxon rank sum test in a univariate analysis. A multivariable analysis was performed to estimate the effect of factors independently associated with colonization by the outbreak strain using a stepwise forward conditional logistic regression. Included in the multivariable analysis were all parameters with a *p*-value ≤ 0.100 in the univariate analysis. The *p*-values for including a variable in the multivariable model was ≤0.05 and for excluding >0.05. Odds ratios (OR) and their 95% confidence intervals (CI 95%) were calculated. All analyses were performed using SPSS (IBM).

## Results

**Fig. 2:**
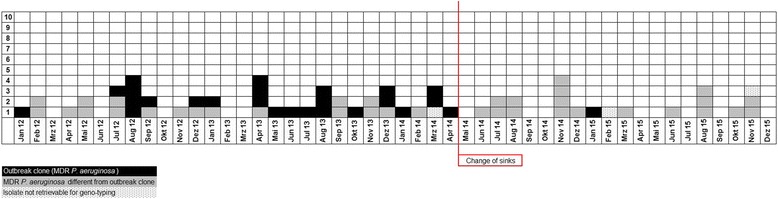
Rate of MDR *P. aeruginosa* in clinical specimens before and after the intervention

Between 1^st^ January, 2012 and 30^th^ April 2014 altogether 135 *P. aeruginosa*-colonized or infected patients were found on the respective ward. The mean length of stay in the outbreak ICU during this time period was 6.34 days. Based on the results of the rep-PCR examination, we found a cluster of 26 patients overall, colonized with the same MDR PAE strain (Fig. 2). This strain showed a phenotypical resistance to acylureidopenicillins, 3^rd^ generation cephalosporins, quinolones and carbapenems. One MDR PAE isolate from March 2014 could not be retrieved for rep-PCR and was excluded from the analysis. The cases were more or less homogeneously scattered over the total study period of 28 months, with a period of 5 months between February and June 2012 during which no MDR PAE were observed (Fig. 2). The typing results and the epidemiological curve suggested a continuous outbreak with a single MDR PAE strain that had lasted since at least January 2012. Hence, we performed environmental examinations of all sinks located in patients’ rooms. Altogether, five sinks were colonized with MDR PAE. Rep-PCR showed that all five isolates were identical to the outbreak strain (Fig. 1).

For five of the 26 cases, sufficient data was not available at the time of the analysis. For the remaining 21 cases (81%), we conducted a case control study using one control per case. Table 1 shows the results of the univariate analysis of all collected parameters for cases and controls. There was no statistically significant difference in age, sex or severity of disease (SAPS II). Among controls, 42.9% (9/21) were colonized with a MDR *P. aeruginosa* different from the outbreak strain. Furthermore, 66.7% (*n* = 14) of the cases and 28.6% (*n* = 6) of the controls had been located in one of the rooms with a sink colonized with the outbreak strain (*p* = 0.062; see Table 1). In the multivariable analysis, stay in a room with a colonized sink and dialysis/hemofiltration showed an increased risk for acquisition of the outbreak strain (Table 2).

**Table 1:** Univariate analysis of characteristics and parameter of cases and controls

Parameter	Cases (*n* = 21)	Controls (*n* = 21)	*p*-value
*P. aeruginosa* infection; % (n)	42.9 (9)	52.4 (11)	0.758
MDR *P. aeruginosa* colonization; % (n)	100% (21)	42.9 (9)	<0.001
SAPS II total (95%Confidence Interval [CI])	50 (37–59)	44 (31–50)	0.147
Colonization site; % (n)			
*Tracheobronchial secretion*	42.9 (9)	57.1 (12)	
*Intraabdominal swab*	9.5(2)	9.5 (2)	
*Rectal*	28.6 (6)	-	
*Wound swab*	9.5 (2)	-	
*Urine*	38.1 (8)	28.6 (6)	
*Blood*	-	4.8 (1)	
Stay in a room with colonized sink; % (n)	66.7 (14)	28.6 (6)	0.062^a^
Contact with MDR colonized patient; % (n)	14.3 (3)	-	
All drainages; % (n)	42.9 (9)	61.9 (13)	0.751
Dialysis or hemofiltration	33.3 (7)	4.8 (1)	0.045^a^
Gastric tube	95.2 (20)	85.7 (18)	0.606
Urinary catheter	100.0 (21)	100.0 (21)	1.000
Tracheal cannula	38.1 (8)	33.3 (7)	1.000
Central venous catheter	90.5 (19)	85.7 (18)	1.000
Leukopenia	14.3 (3)	14.3 (3)	1.000
Immunosuppression	38.1 (8)	9.5 (2)	0.067^a^
Septic chock	19.0 (4)	28.6 (6)	0.719
Surgery patient	85.7 (18)	90.5 (19)	1.000
Neurosurgery patient	14.3 (3)	23.8 (5)	0.697
Age (years)	71.0 (55.2–78.1)	75.5 (63.4–83.4)	0.120
LOS before colonization (days)	16 (11–34)	10 (5–28)	0.190
Length of stay overall (days)	38 (23–54)	30 (10–46)	0.170
Stay in single room (days)	0 (0–5)	0 (0–9)	1.000
Stay in shared room (days)	12 (6–30)	4 (0–16)	0.031^a^
Penicillin; % (n)	42.9 (9)	23.8 (5)	0.362
Cephalosporin; % (n)	42.9 (9)	23.8 (5)	0.362
Carbapenem; % (n)	42.9 (9)	28.6 (6)	0.520
Quinolone; % (n)	52.4 (11)	23.8 (5)	0.111
Aminoglycoside; % (n)	28.6 (6)	4.8 (1)	0.093^a^
Glycopeptide; % (n)	28.6 (6)	19.0 (4)	0.719
Antibiotic inhalation; % (n)	33.3 (7)	9.5 (2)	0.130

Continuous data is displayed as median (interquartile range) and categorial data as percentage (number)

*SAPS* Simplified Acute Physiology Score (score for the severity of disease), *LOS* length of stay

^a^parameter was included in the multivariable regression analysis

**Table 2:** Results of the multivariable regression analysis

Parameter	Odds ratio	*P*-value	95%Confidence interval
Stay in a room with colonized sink	**11.229**	**0.007**	**1.920–65.687**
Dialysis or hemofiltration	**21.874**	**0.020**	**1.628–293.910**
Immunosuppression	7.868	0.057	0.942–65.736

Included in the analysis were stay in a room with colonized sink, dialysis or hemofiltration, immunosuppression, aminoglycosides. Independent factors are displayed in bold

*CI* confidence interval

In order to prevent further spread of the outbreak strain, we replaced the traps of the affected sinks and implemented single use hemofiltration bags and emptying was performed outside patient rooms. Furthermore, we performed additional training in hand hygiene and education of potential transmission routes for the ward’s staff. As a follow up, we analyzed the detection rate and the incidence density of MDR *P. aeruginosa* in clinical specimens before and after the intervention (replacement of colonized sinks/single use bags) (Table 3). After the intervention, a significant reduction was seen in the incidence density as well as the detection rate for the outbreak strain. A decreased detection rate in overall MDR *P. aeruginosa* clinical specimens was observed after the intervention but also an increased incidence density of clinical specimens.

**Table 3:** MDR *P. aeruginosa* incidence density and detection rate before and after intervention

	Before Intervention (28 months)	After Intervention (20 months)	*P*-value
Patient days	21,324	10,874	0.255
Clinical specimen examinations	*n* = 3,469	*n* = 5,388	0.008
Outbreak strain incidence density (number/1,000 patient days)	1.22	0,18	0.001
Outbreak strain detection rate (number/1,000 clinical specimen)	7.49	0.37	<0.001
MDR PAE incidence density (number/1,000 patient days)	4.13	7.45	<0.001
MDR PAE detection rate (number/1,000 clinical specimen)	25.37	15.03	<0.001

Incidence density was defined as number of specimens per 1000 patient days. Detection rate was defined as number of specimens per 1000 clinical specimen. Clinical specimens were obtained from tracheobronchial secretions, intraabdominal swabs, rectal swabs, wound swabs, urine cultures and blood cultures. Period prior to intervention was from 01/2012 to 04/2014, a period

## Discussion

Patient colonization, or infection with MDR *P. aeruginosa* in intensive care units is a common phenomenon [12]. It is important to minimize the likelihood of transmission to or between ICU patients. Intensive care units are high-risk areas for nosocomial infections. Hence, ICUs require particular infection prevention measures (different from standard care wards) in order to take into account the elevated risk of infection and transmission associated with ICU-specific working procedures (e.g. ventilator support, central-line catheters). Within the scope of an outbreak investigation due to MDR PAE in an ICU, we found evidence of a transmission route associated with working procedures at sink, in particular the use of the sinks for grooming the patients. After reducing the procedures associated with sinks in patient rooms by using single use octenidine wash cloths [7] and the instruction to empty ultra-filtrate bags of the hemofiltration in a sink outside the patient room. After the implementation the outbreak strain was detected less frequently. The increase of incidence density for all MDR PAE must be seen as a result of a post outbreak increase of microbiological screening activities. This is underlined by the significant decline in the detection rate per clinical specimen and the significantly more frequent microbiological examinations after the intervention.

**Fig. 3:**
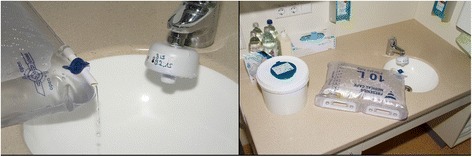
Example of ultra-filtrate bag and contact with sink in patient room

Contaminated sinks or faucets have been described as sources for *P. aeruginosa* outbreaks in the past [1, 2, 4, 11, 19, 20]. In many outbreaks, contaminated washbasins served as continuous sources. From there, *P. aeruginosa* is often spread further from person to person through direct contact. Our data supports this finding. Two thirds of our cases had a stay within a room with a colonized sink. Furthermore, one third of the patients had renal replacement therapy, such as dialysis or hemofiltration. In line with national recommendations, the suggested procedure in our hospital is to dispose ultra-filtrate bags via waste rooms. While investigating on site, we found that the ultra-filtrate bags from the dialysis or hemofiltration machines where used multiple times and emptied in the nearest sink (Fig. 3). The ultra-filtrate bags had a filling volume of 10 l and were changed five times per 24 h on average. The strong statistical association of renal replacement therapy with the outbreak strain could therefore reveal an as yet not described transmission route. However, two-thirds of the case patients did not receive renal replacement therapy. Therefore, other transmission routes, such as direct person-to-person contact, were most likely also involved. The improvement of hand hygiene through focused training was a key element of our outbreak control. Also splash of water from the sink into hands of staff and contamination of medical must be taken into account. As an outbreak control measure, we changed all contaminated traps and recommended to switch to single-use ultra-filtrate bags to limit the procedures associated with sinks located in patient rooms. However, within the subsequent 20 months after the reduction of working processes associated with sinks in patient rooms, the clinical detection of the outbreak isolate as well as the incidence density of MDR *P. aeruginosa* declined significantly. A study by Hopmann et al. in the Netherlands showed a reduction of the MDRO rate in an ICU after removing sinks from patient rooms [9].

In the univariate analysis, prolonged stay in a shared room was associated with the colonization by the outbreak strain. This did not turn out to be an independent factor in the multivariable analysis. However, currently there is an ongoing discussion whether single rooms in ICUs have the potential to reduce nosocomial infections [16]. As only few of the patients analyzed were infected with *P. aeruginosa,* our contribution to this discussion is limited.

## Conclusion

Sinks in patient rooms in ICUs can be potential drivers of MDR *P. aeruginosa* outbreaks. We were able to show that reducing work processes involving sinks in patient rooms result in reduced MDR *P. aeruginosa rates*. ICUs with high rates of *P. aeruginosa* should consider eliminating work processes involving sinks in proximity to patients.

## Abbreviations

CI: Confidence intervals
CVC: Central venous catheter
ICU: Intensive care unit
MDR PAE: Multidrug-resistant Pseudomonas aeruginosa
MDR: Multidrug-resistant
MDRO: Multidrug resistant organisms
OR: Odds ration
P. aeruginosa: Pseudomonas aeruginosa
rep-PCR: Repetitive element-based polymerase chain reaction
